# Prognostic impact of a novel gene expression profile classifier for the discrimination between metastatic and non-metastatic primary colorectal cancer tumors

**DOI:** 10.18632/oncotarget.22591

**Published:** 2017-11-21

**Authors:** María Laura Gutiérrez, Luis Antonio Corchete, María Eugenia Sarasquete, María del Mar Abad, Oscar Bengoechea, Encarna Fermiñán, María Fernanda Anduaga, Sofía del Carmen, Manuel Iglesias, Carmen Esteban, María Angoso, Jose Antonio Alcazar, Jacinto García, Alberto Orfao, Luis Muñoz-Bellvís, José María Sayagués

**Affiliations:** ^1^ Cytometry Service-NUCLEUS, Cancer Research Center, IBMCC-CSIC/USAL, Department of Medicine, University of Salamanca, Institute of Biomedical Research of Salamanca, Biomedical Research Networking Centre Consortium-CIBER-CIBERONC, Salamanca, Spain; ^2^ Cancer Research Center and Service of Hematology, University Hospital of Salamanca, Salamanca, Spain; ^3^ Department of Pathology, University Hospital of Salamanca, Salamanca, Spain; ^4^ Genomics Unit, Cancer Research Center, IBMCC-CSIC/USAL, Salamanca, Spain; ^5^ Service of General and Gastrointestinal Surgery, Institute of Biomedical Research of Salamanca, Salamanca, Spain

**Keywords:** sporadic colorectal cancer, metastatic, non-metastatic, primary tumor, gene expression profile

## Abstract

Despite significant advances have been achieved in the genetic characterization of sporadic colorectal cancer (sCRC), the precise genetic events leading to the development of distant metastasis remain poorly understood. Thus, accurate prediction of metastatic disease in newly-diagnosed sCRC patients remains a challenge. Here, we evaluated the specific genes and molecular pathways associated with the invasive potential of colorectal tumor cells, through the assessment of the gene expression profile (GEP) of coding and non-coding genes in metastatic (MTX) vs. non-metastatic (non-MTX) primary sCRC tumors followed for >5 years. Overall, MTX tumors showed up-regulation of genes associated with tumor progression and metastatic potential while non-MTX cases displayed GEP associated with higher cell proliferation, activation of DNA repair and anti-tumoral immune/inflammatory responses. Based on only 19 genes a specific GEP that classifies sCRC tumors into two MTX-like and non-MTX-like molecular subgroups was defined which shows an independent prognostic impact on patient overall survival, particularly when it is combined with the lymph node status at diagnosis. In summary, we show an association between the global GEP of primary sCRC cells and their metastatic potential and defined a GEP-based classifier that provides the basis for further prognostic stratification of sCRC patients who are at risk of distant metastases.

## INTRODUCTION

Despite significant progress made in the last decades, sporadic colorectal cancer (sCRC) still remains one of the most frequent causes of cancer death in the Western world [1]. Most sCRC deaths are caused by metastatic dissemination of primary tumors, mainly into the liver [2]. Although sCRC is amongst the most well-characterized solid tumors at the molecular level [1], the specific genes and molecular mechanisms involved in the metastatic process of sCRC, still remain to be fully identified.

For many years, it has been postulated that the genetic/biological markers associated with the ability of sCRC to invade distant tissues already resides within primary tumor cells [2, 3]. Thus, we and others have recently shown that specific genomic alterations found in metastatic sCRC –e.g. del(17p) and del(22q)- are shared by primary tumors and their paired liver metastatic samples [2, 4–7], while absent in non-metastatic sCRC [8, 9]; interestingly, such genomic alterations of sCRC were also shown to be closely associated with unique gene expression profiles (GEP) [6, 7, 10]. Of note, the impact of such sCRC metastasis-associated genetic changes and GEP depends both on the alteration of specific protein-coding genes and the aberrant expression profiles of their post-transcriptional regulators (e.g. miRNAs) [10, 11]. Thus, simultaneous assessment of the GEP of both mRNA and non-coding (nc)RNA, might further contribute to better understand the molecular pathways involved in sCRC, via more accurate definition of those GEP that are responsible for the metastatic potential of primary CRC tumor cells.

To date, few studies have combined global mRNA and miRNA GEP analyses in sCRC [12–16]. In such studies, altered GEP, molecular signaling pathways and/or mRNA-miRNA interactions associated with colorectal carcinogenesis have been explored, leading to the identification of mRNA/miRNA GEP associated with prognosis of sCRC patients and metastatic dissemination of sCRC tumors [15–17]. In two of these studies, mRNA/miRNA GEP associated with different stages of both normal and tumor (e.g. low-grade intraepithelial neoplasia, high-grade intraepithelial neoplasia, and adenocarcinoma) human colonic development allowed identification of two distinct molecular signatures – a 55- [15] and a 12-gene GEP [16] –associated with significantly different sCRC survival rates. In turn, Wang *et al.* [17] identified 6 miRNAs involved in regulatory loops of transcription factors that might play a role in the development of sCRC metastasis, based on the analysis of independent sets of publicly available mRNA and miRNA GEP data on (distant and/or locally) invasive primary sCRC tumors vs. non-metastatic sCRC tissues. However, in none of the above reports the overall GEP of coding plus non-coding RNA were simultaneously assessed in primary tumor cells from metastatic vs. (long-term) non-metastatic sCRC patients, in order to better identify sCRC primary tumors at risk of harboring distant metastases.

Here, we evaluated the overall GEP of primary sCRC tumors from 48 newly-diagnosed patients (23 metastatic tumors vs. 25 non-metastatic tumors) with a long follow-up based on simultaneous assessment of the transcriptomics profile of both coding and non-coding RNA genes -including mRNA, miRNA, small nucleolar and large intergenic RNAs-. Our major goal was to identify GEPs among primary sCRC tumors that could contribute to differentiate, already at diagnosis, between metastatic and non-metastatic patients, and to improve our current understanding of the genomic landscape of sCRC metastasis.

## RESULTS

### GEP of MTX vs. non-MTX sCRC primary tumor samples

Supervised analysis of the GEP of primary MTX (*n* = 23) vs. non-MTX (*n* = 25) sCRC tumors showed 12/189 (6%) and 113/189 (60%) -mRNA and (nc)RNA- genes to be exclusively expressed in MTX and non-MTX tumors -vs. non-tumoral colorectal tissues (*n* = 9)-, respectively; another 11/189 (6%) genes were commonly deregulated in both groups of sCRC tumors. The remaining 53 genes displayed significantly different expression levels between both types of sCRC tumors (false discovery rate, FDR ≤ .05) but they did not show differential expression vs. non-tumoral colorectal tissues (Supplementary Table 1). Of note, within MTX sCRC tumors similar GEP were found between cases that presented with metastasis already at diagnosis and these that developed metastatic cancer afterward (Supplementary Figure 1A). Consequently, these two subgroups of MTX tumors were considered together hereafter.

Tumor-associated genes which were most overexpressed in MTX vs. non-MTX primary tumors, included genes involved in cell adhesion, extracellular matrix (ECM) and/or tissue remodeling processes (e.g. the SPP1, SFRP4, COMP genes), and progression and migration of tumor cells (also known to be typically altered in sCRC, such as the SRPX2, SALL4 genes and the non-coding FER1L4 and miR-21 genes). Metastatic sCRC tumors also showed high-level down-regulation of tumor suppressor miRNAs, including members of the miR-378 family (miR-378, miR-378c, d and e) and miR-422a, together with loss of expression of the ADGRG7 gene involved in cell adhesion, and genes previously described to be silenced during progression and/or invasion of gastrointestinal tumors (e.g. the PIGR and ADH1B genes). In turn, those genes most strongly overexpressed in non-MTX sCRC tumors included genes involved in tissue regeneration (e.g. REG1A and REG3A), MMP3, pro-inflammatory chemokine ligands (CXCL5 and CXCL3), and the miR-3175 and miR-25* genes, all of which have been previously associated with malignant transformation of gastrointestinal tissues; this was associated with abnormally low expression levels of genes coding for muscle-related proteins that support cell migration and invasion (e.g. TAGLN, MYH11, DES and CNN1), and genes typically silenced in sCRC (e.g. FABP4 and AKAP12) (Table 1).

**Table 1:** Most differentially deregulated mRNA and small non-coding RNA transcripts in metastatic (*n* = 23) vs. non-metastatic sCRC primary tumors (*n* = 25) vs. non-tumoral colorectal tissues (*n* = 9)

Gene Name	Gene ID	MTX vs. non-MTX sCRC (Fold change)	MTX sCRC vs. non-tumoral (Fold change)	Non-MTX sCRC vs. non-tumoral (Fold change)	Chromosomal band
***Up-regulated transcripts in sCRC vs. normal CR tissue***					
REG1A	ENSG00000115386	**–9.4**	NS	18.1	2p12
REG3A	ENSG00000172016	**–8.5**	NS	8.6	2p12
CXCL5	ENSG00000163735	**–4.1**	NS	6.6	4q13.3
MMP3	ENSG00000149968	**–3.9**	4.1	15.6	11q22.2
SPP1	ENSG00000118785	**3.7**	6.7	NS	4q22.1
hsa-miR-3175	MI0014209	**–3.6**	NS	6.5	15q26
hsa-miR-25^*^	MI0000082	**–3.6**	NS	5.6	7q22.1
SFRP4	ENSG00000106483	**3.2**	5.3	NS	7p14.1
COMP	ENSG00000105664	**3**	6.0	2.0	19p13.1
SRPX2	ENSG00000102359	**2.7**	13.8	5.2	Xq22.1
CXCL3	ENSG00000163734	**–2.6**	5.0	13.0	4q13.3
hsa-miR-21	MI0000077	**2.1**	3.8	NS	17q23.1
SALL4	ENSG00000101115	**2.0**	3.3	NS	20q13.2
FER1L4	ENSG00000088340	**2.1**	2.9	NS	20q11.2
***Down-regulated transcripts in sCRC vs. normal CR tissue***					
FABP4	ENSG00000170323	**6.1**	NS	–15.1	8q21
PIGR	ENSG00000162896	**–4.2**	–10.7	NS	1q32.1
hsa-miR-422a	MI0001444	**–3.4**	–6.4	NS	15q22.3
hsa-miR-378c	MI0015825	**–3.3**	–7.0	–2.1	10q26.3
hsa-miR-378	MI0000786	**–2.9**	–5.2	NS	5q32
hsa-miR-378d	*	**–2.8**	–6.6	–2.3	*
TAGLN	ENSG00000149591	**2.8**	NS	–6.7	11q23.3
MYH11	ENSG00000133392	**2.7**	–4.0	–10.7	16p13.1
DES	ENSG00000175084	**2.6**	NS	–8.2	2q35
AKAP12	ENSG00000131016	**2.5**	NS	–6.3	6q25.1
ADH1B	ENSG00000196616	**2.4**	–7.6	–18.1	4q23
ADGRG7	ENSG00000144820	**–2.3**	–3.2	NS	3q12.2
hsa-miR-378e	MI0016750	**–2.2**	–4.3	NS	5q35.1
CNN1	ENSG00000130176	**2.2**	NS	–8.9	19p13.2

*FDR* < .05; sCRC sporadic colorectal cancer; MTX: metastatic colorectal cancer; non-MTX: non-metastatic colorectal cancer; hsa-miR: human micro-RNA; NS: no statistically significant differences observed (*p* > .05); ^*^miRNA transcripts with various annotated stem-loop sequences located in chromosomes 8q22.1 and 4p16.2.

### Functional characterization of GEP in MTX vs. non-MTX sCRC primary tumors

**Figure 1:**
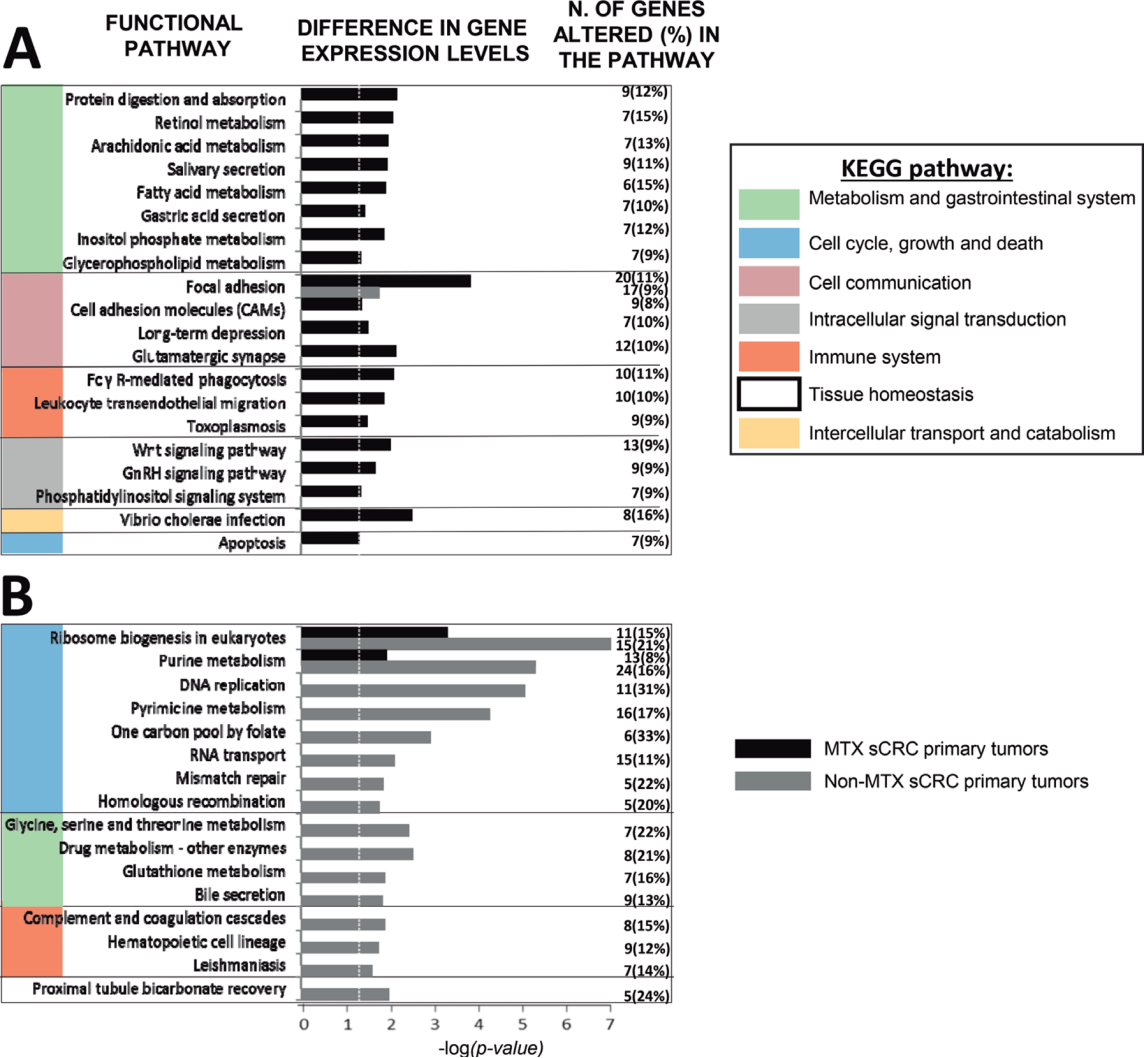
Most representative molecular (KEGG) pathways differentially deregulated in metastatic (MTX; panel **A**) and non-metastatic (non-MTX; panel **B**) primary sCRC tumors as defined by their GEP for both coding and non-coding RNAs (*n* = 48; *p* < .05). GnRH: Gonadotropin-releasing hormone. Dotted vertical line indicates corrected *p-value* < .05.

Functional enrichment analysis of tumor-associated GEPs altered in MTX vs. non-MTX sCRC primary tumors revealed specific molecular pathways to be differentially deregulated in both groups of tumors (Figure 1; Supplementary Table 2). Thus, MTX sCRC tumors commonly showed down-regulation of genes involved in fatty acid and phospholipid metabolism, and the gonadotropin-releasing hormone (GnRH) and phosphatidylinositol signaling pathways; this included down-regulation of the PLA2G2A, PLA2G4, PLCD1 and PLCE1 phospholipase genes, and the PIP5K1B, ITPKA and IMPA2 genes, among other genes. In contrast to non-MTX sCRC cases, MTX tumors also displayed altered expression of genes involved in cell adhesion molecules (CAMs) -including focal cell-to-cell and intercellular adhesion molecules-, in protein digestion and absorption, and in the glutamatergic synapse signaling pathways, together with genes involved in intercellular transport via endocytosis; this included overexpression of several members of the collagen gene family (e.g. COL5A1, COL5A2, COL9A3, COL11A1), CDH3, IBSP, COMP and ATPase proton transporter genes (e.g. ATP6V0D1, ATP6V1C2 and ATP6V1F), together with decreased expression of the CLDN8 and CLDN23 claudins and the NECTIN3 gene, among other genes. In parallel, genes that participate in pro-inflammatory and/or protective immune functions were also downregulated in MTX sCRC such as pro-inflammatory effector genes involved in FcγR-mediated phagocytosis and/or the toxoplasmosis pathways (e.g. the PIP5K1B, CRK, PLA2G2A, PLA2G4A, PLPP1 and PLPP3 genes) and chemokines responsible for leucocyte migration (e.g. CXCL12). On top of all the above, MTX tumors also showed up-regulation of the WNT signaling pathway, together with down-regulation of apoptosis, as reflected by an increased expression of the WNT11, DKK1, AXIN2 and NKD2 wingless-related genes, and the inhibition of the FAS, CASP7 and TNFSF10 programmed cell-death associated genes, among other genes (Figure 1).

In turn, non-MTX sCRC also displayed a uniquely altered GEP consisting of overexpression of a broad number of genes related to cell proliferation and DNA repair: several ribonucleoproteins (e.g. RPP25, RPP40, POP, NOP56 and NOP58), members of the minichromosome maintenance complex (e.g. MCM2, MCM4 and MCM7), DNA/RNA polymerase genes (e.g. POLA1, POLD2, POLE2, POLR1B and POLR1C), replication factors (e.g. RFC4 and RFC5) and the PCNA and TYM2 genes; in addition, non-MTX tumors showed increased expression levels of genes involved in the homologous recombination process such as BRCA, BLM, the exonuclease EXO1 and the RAD54L and RAD54B helicases. Noteworthy, non-MTX sCRC tumors also displayed altered GEP related to the inflammatory response consisting of: i) upregulation of complement regulatory proteins (e.g. C4BPB and CD46) and genes involved in the coagulation signaling cascade (e.g. overexpression of PLAU and decreased levels of the PLAT gene); ii) decreased expression of C7 and the CD21 complement receptor 2; iii) inhibition of hematopoietic differentiation pathways with altered CD14, CD1d and CD36 gene expression levels, and; iv) overexpression of the low affinity FCGR3A and FCGR3B immunoglobulin G receptor genes, the CD44 cell adhesion/signaling molecule and interleukin IL1A, together with v) down-regulation of the A2M and the ANPEP genes (Figure 1).

### GEP that discriminate between MTX and non-MTX sCRC primary tumors

**Figure 2:**
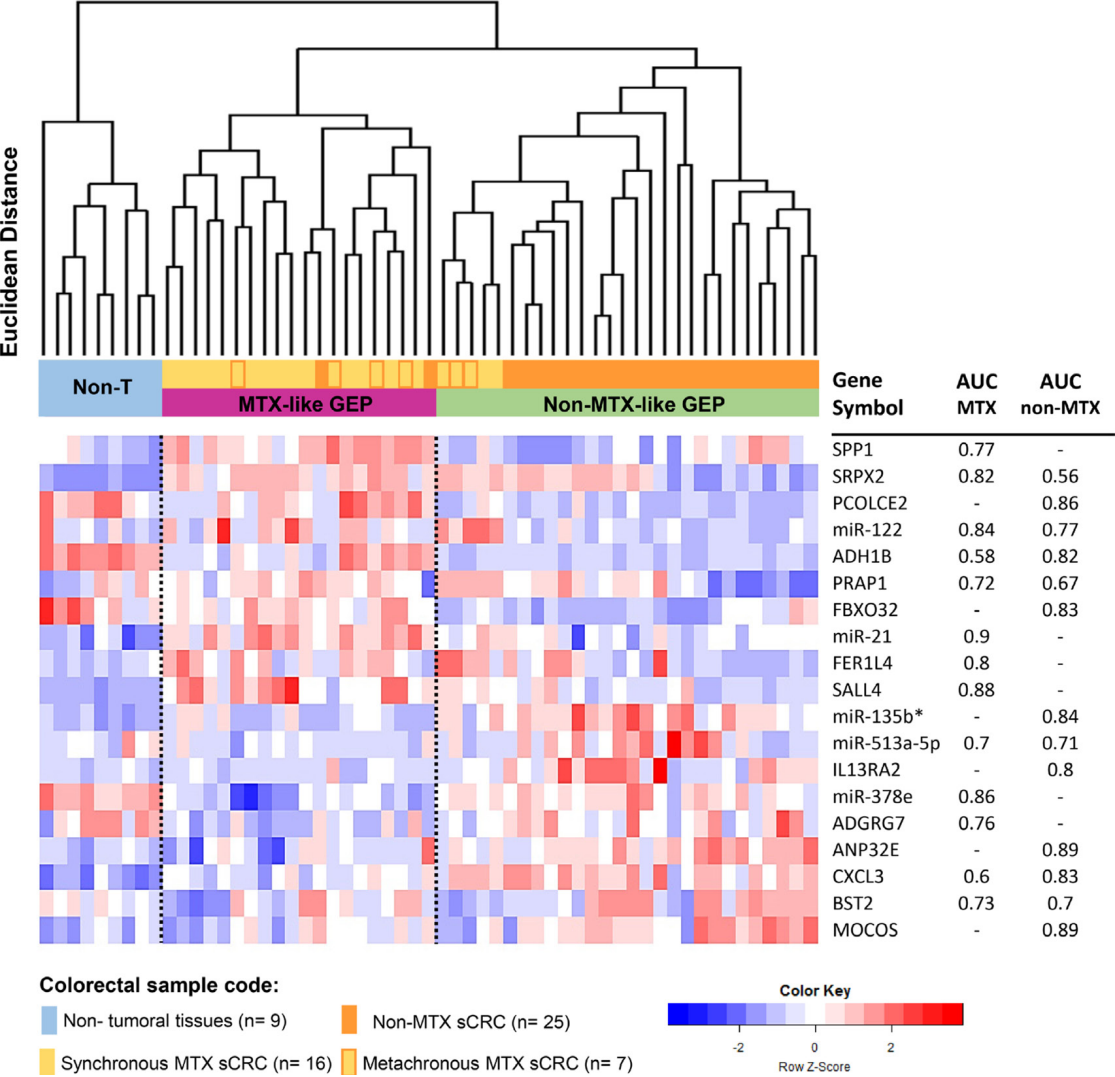
Association between sCRC tumor specific gene expression profiles (GEP) and the metastatic behavior of the tumor Unsupervised hierarchical clustering analysis and the corresponding GEP heatmap show a clear different profile between the two groups of sCRC tumors vs. non-tumoral colorectal tissues (*n* = 9; color coded as light blue) based on the combination of 19 selected coding (mRNA) and non-coding (small nuclear and microRNA) genes: a non-metastatic-like GEP group of tumors (non-MTX-like GEP, *n* = 27; colored green) which predominantly included non-metastatic sCRC cases (colored orange) and a few metachronous metastatic sCRC primary tumors (colored yellow with orange frames) vs. a metastatic-like GEP group (MTX-like GEP, *n* = 21; colored purple) mostly consisting of metastatic primary sCRC cases (colored yellow). Area under the curve (AUC) values derived from ROC curve analysis for those individual 19 genes selected by the prediction algorithms, which better contributed to discriminate between MTX and non-MTX tumoral groups vs. non-tumoral colorectal tissues (*n* = 23 vs. *n* = 25 vs. *n* = 9, respectively), are displayed in the columns in the right.

From all RNA transcripts found to be differentially expressed in MTX vs. non-MTX sCRC tumors, a subset of only 19 altered genes allowed clear-cut discrimination between the two groups (MTX vs. non-MTX) of sCRC tumors with an accuracy of 95% (Figure 2) and 97% (Supplementary Figure 1B), in the global series and when patients who displayed liver metastases already at diagnosis were excluded from the analysis, respectively. Thus, unsupervised hierarchical clustering analysis (HCA) of both tumor and normal colorectal tissues, based on these 19 genes, differentiated between non-tumoral tissues and two well-defined subgroups of sCRC tumors: 1) a MTX-like sCRC subgroup consisting of 18/23 MTX sCRC primary tumor samples (78%) plus 2/25 non-MTX sCRC primary tumor specimens (8%), and 2) a non-MTX-like sCRC subgroup that included 23 non-MTX (92%) samples that clustered together with 5 MTX sCRC primary tumors (2 synchronous and 3 metachronous). Interestingly, although those 2 non-MTX samples clustering in the MTX-like GEP subgroup did not display distant liver metastasis nor detectable lymph node involvement after >10 years follow-up, they developed a locoregional relapse in one case and a *de novo* urothelial carcinoma in the other patient. The GEP-classifier here proposed included the following 19 altered genes: 1) overexpression in both groups of sCRC primary tumors (vs. non-tumoral colorectal tissues) of the SRPX2 and CXCL3 genes, and down-regulation of ADH1B; 2) up-regulation of SPP1, miR-21, FER1L4 and SALL4, together with down-regulation of miR-378e and ADGRG7, in MTX tumors (vs. non-tumoral colorectal tissues); 3) up-regulation (vs. non-tumoral colorectal tissues) of the IL13RA2, miR-135b*, ANP32E and MOCOS genes, plus down-regulation of FBXO32 and PCOLCE2 in non-MTX tumors; and 4) abnormally high expression of the miR-122 and PRAP1 genes together with down-regulation of BST2 and miR-513a-5p observed in MTX vs. non-MTX sCRC cases (but not vs. non-tumoral colorectal tissues). All individual 19 genes showed an acceptable ability to discriminate, with area under the curve (AUC) values above 0.7 in receiver operating characteristic (ROC) curve analysis (Figure 2).

### Prognostic impact of the GEP of MTX vs. non-MTX sCRC primary tumors

**Figure 3:**
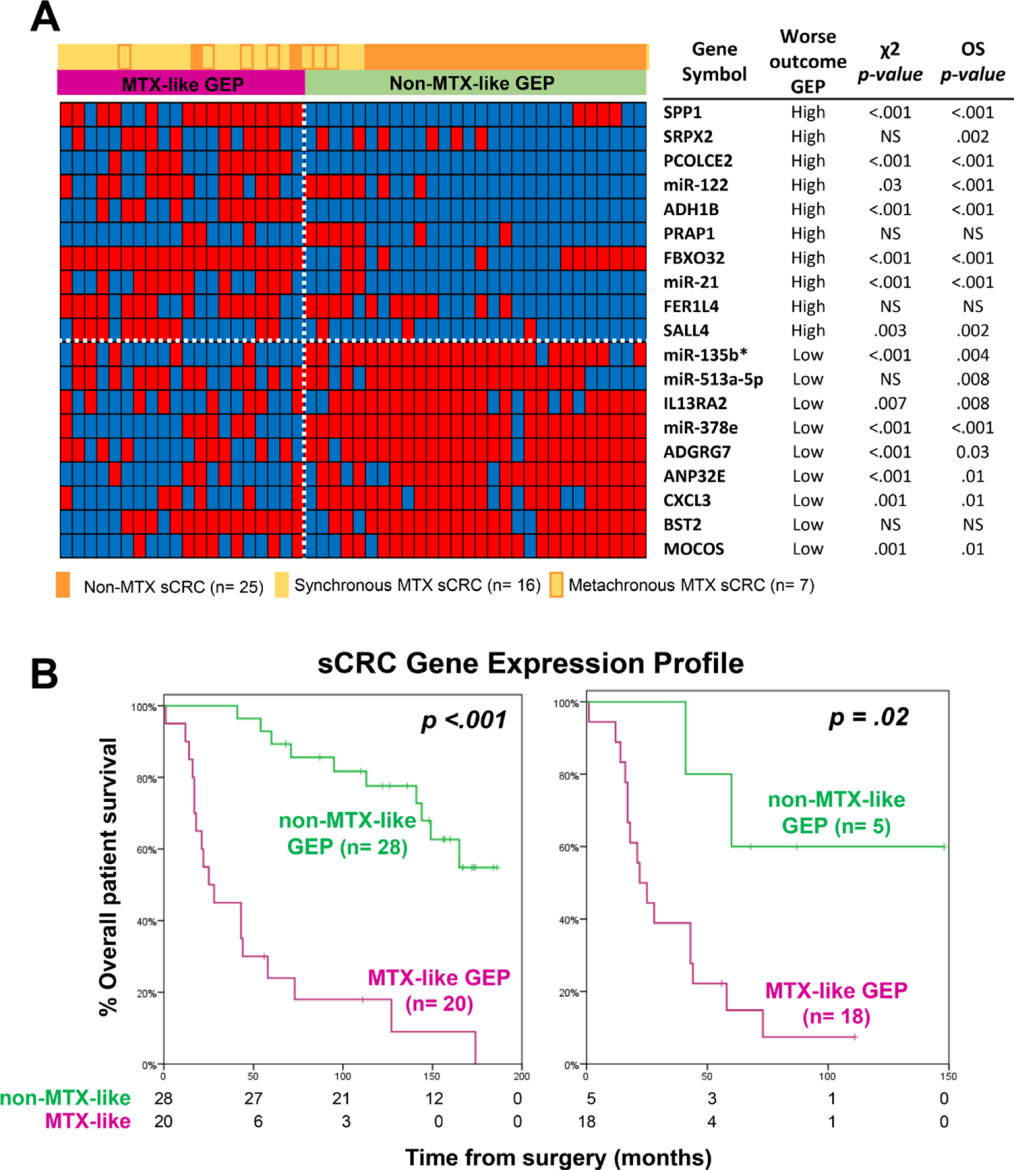
Prognostic impact of the GEP classifier based on those 19 coding plus non-coding genes that better differentiated between metastatic and non-metastatic sCRC (**A**) Heatmap of categorized (dichotomized) gene expression levels -low (blue) versus high (red)- observed among metastatic (*n* = 23) vs. non-metastatic (*n* = 25) sCRC tumors, and its association with the two GEP tumor subgroups shown in Figure 2. In the right, the impact of the expression levels of each of the discriminating genes on overall survival (OS) is shown. (**B**) Prognostic impact of the overall GEP for those 19 discriminating genes selected, on overall survival for both the whole series (*n* = 48; left panel) and patients with metastatic liver disease (*n* = 23; right panel).

From those 19 genes included in the GEP-classifier for MTX vs. non-MTX sCRC tumors, higher expression levels of the SPP1, SRPX2, PCOLCE2, miR-122, ADH1B, PRAP1, FBXO32, miR-21, FER1L4 and SALL4 genes, together with lower expression levels of the miR-135b*, miR-513a-5p, IL13RA2, miR-378e, ADGRG7, ANP32E, CXCL3, BST2 and MOCOS genes, were all associated with a significantly shorter overall survival (OS) for the whole patient series (*n* = 48; *p* ≤ .03; Figure 3A). Thereby, the overall 19-gene classifier also showed a strong prognostic impact on patient OS, both for the whole patient series (*p* < .001) and also specifically for MTX sCRC patients, among whom it identified a small subgroup of metastatic cases (*n* = 5) with a significantly better outcome (*p* = .02; Figure 3B). Other disease features that showed an adverse impact on patient OS (*p* ≤ .05) included: increased (>7.5 ng/ml) carcinoembryonic antigen (CEA) serum levels, larger tumor sizes, histologically poorly-differentiated tumors, lymph node involvement and metastatic liver disease at diagnosis. Multivariate analysis of prognostic factors for OS showed that the 19-gene classifier here proposed, together with the presence of lymph node involvement at diagnosis, were the only two independent variables (*p* < .001); thus, the combination of these two variables allowed stratification of sCRC patients already at diagnosis into low- (patients displaying a non-MTX-like GEP, independently of their lymph node infiltration status; *n* = 28), intermediate- (MTX-like GEP without lymph node involvement; *n* = 8) and high-risk (MTX-like GEP associated with lymph node involvement; *n* = 12) subgroups, with significantly different OS rates at 5-years and a high potential prognostic ability (high concordance probability with a Harrell´s c-index of 0.8): 89% ± 6% vs. 60% ± 18% and 0% ± 0%, respectively (*p* < .001; Table 2 and Supplementary Figure 2).

**Table 2:** Univariate and multivariate analysis of prognostic factors for overall survival in sCRC patients (*n* = 48)

Patient tumor features	N. of cases (%)	Median OS (months)	Univariate analysis			Multivariate analysis		
			Hazard Ratio	95% CI	*p*-value	Hazard Ratio	95% CI	*p*-value
**Tumor size**					**.001**			NS
≤5 cm	32 (67%)	149	-	-				
5.1–6.9 cm	4 (8%)	16	6.6	2.1–21				
>7 cm	12 (25%)	73	1.5	0.6–3.6				
**CEA serum levels**					**.005**			NS
≤7.5 ng/ml	28 (58%)	165	-	-				
>7.5 ng/ml	20 (42%)	41	2.82	1.3–6				
**Histopathological grade**					**.007**			NS
Well-differentiated	29 (60%)	165	**-**	**-**				
Moderately/poorly-differentiated	19 (40%)	44	2.8	1.2–6				
**Lymph node involvement**					**<.001**			**<.001**
N0	33 (69%)	165	**-**	**-**				
N ≥ 1	15 (31%)	25	7.2	3.1–17		**6.54**	**2.5–17**	
**Extranodal metastasis at diagnosis**					**<.001**			NS
No	35 (73%)	165	-	-				
Yes	13 (27%)	25	8.3	3.3–21.7				
**TNM stage at diagnosis**					**<.001**			NS
I–IIIC	35 (73%)	165	**-**	**-**				
IV	13 (27%)	25	8.3	3.2–21.7				
**sCRC GEP-classifier**					**<.001**			**<.001**
Non-MTX-like	28 (58%)	NR	-	-				
MTX-like	20 (42%)	25	7.5	3.3–17		**7**	**2.9–17.1**	

CI: confidence interval; NR: not reached; NS: no statistically significant differences found (*p* ≤ .05); CEA: carcinoembryonic antigen.

## DISCUSSION

Currently, prognostic stratification of sCRC still relies to a large extent on tumor size and dissemination at diagnosis, (TNM) staging system [18]. However, such classification does not predict for the potential development of subsequent metastatic disease, particularly for patients diagnosed with early stage sCRC [19]. Despite important advances have been made in the characterization of the molecular features of sCRC [1], the precise genetic events that lead to the development of distant metastasis and prediction of tumor behavior, still remain unclear [18]. In order to gain insight into the specific genes and molecular pathways responsible for the metastatic potential of primary colorectal tumor cells, here we analyzed the global GEP of both coding and non-coding RNA of MTX vs. (long-term) non-MTX sCRC primary tumors. Our major goal was to identify new predictors, that once combined to other conventional prognostic factors, might improve the identification, already at diagnosis, of sCRC patients who are at risk of harboring distant (e.g. liver) metastases.

Overall, our results show that once compared to non-MTX sCRC, MTX primary tumors frequently show up-regulation of genes associated with tumor progression, invasion and metastatic capacity, including genes directly involved in cell-cell and cell-matrix adhesion, ECM degradation and remodeling, cell proliferation, motility and angiogenesis [20–24]. In contrast, genes linked to metabolic and intracellular signaling pathways (i.e: the fatty acid, retinol, glycerophospholipid, GnRH, arachidonic acid and the phosphatidylinositol) related to inflammation [25–27] and cell migration [28, 29], were down-regulated among MTX vs. non-MTX sCRC primary tumor samples. This included down-regulation of genes associated with FcγR-mediated phagocytosis and leucocyte migration [25, 30], together with overexpression of VEGFA [31], suggesting that the migration and establishment of colorectal tumor cells at distant (i.e: metastatic) sites might only occur in a microenvironment depleted of pro-inflammatory signals provided by e.g. locally recruited immune cells in response to injury [32]. Such inhibitory effects on the immune system would contribute to down-regulation of apoptosis and epithelial-cell differentiation and growth signals, as found among our MTX sCRC primary tumors, via down-regulation of genes involved in retinoid signaling (e.g: RARRES1 and RARRES3) [20].

In contrast to MTX tumors, functional enrichment of non-MTX sCRC tissues showed inhibition of those genes associated with invasive sCRC tissues such as ACTA2 [33], together with up-regulation of tumor suppressor genes (e.g. miR-3178) [34]. Moreover, non-MTX sCRC tumors showed up-regulation of multiple genes involved in nucleic acid metabolism and processing pathways, suggesting a higher tumor cell proliferation, as also found previously by others [15, 35] at early stages of colorectal carcinogenesis (e.g: low-grade intraepithelial neoplasia and/or in stage I sCRC). In addition, non-MTX tumors were characterized by up-regulation of genes involved in maintaining of genomic stability including overexpression of multiple DNA damage repair genes (i.e: BRCA2, BLM, RAD54, RAD54B, ANP32E, mIR-513a-5p, CHEK1, INTS7, FANCI and FANCB). Altogether, this might also contribute to explain why non-MTX sCRC primary tumors, in addition to a high proliferation and increased DNA repair GEP, also showed increased expression of cell death and apoptosis-associated genes, including up-regulation of the IL1A and/or miR-513a-5p apoptosis-inducer genes [36, 37]. In turn, non-MTX sCRC tissues, might also be more vulnerable to the immune system (i.e: immunogenic) than MTX tumors, which might recognize better their tumor-associated antigens and restrict their growth [38], as reflected by an increased activation of the immune and inflammatory responses observed here for non-MTX vs. MTX sCRC tumors, due to e.g. up-regulation of the REG1A and REG3A genes [39], the complement and coagulation pathways, and macrophage activity (reflected here by up-regulation of the IL1A, CD44, and APOC1 genes, and the leishmaniasis and phagosome-signaling pathways) [25, 37, 40–42], together with increased expression levels of several chemokines (e.g: CXCL1-3, CXCL5-6, CXCL8-11 and CXCL16 and CCL20) [43] observed among the former cases. Altogether, these results suggest that despite non-MTX sCRC tumors display a higher cell proliferation genomic profile, this is associated with an increased DNA repair and activation of the anti-tumoral immune and inflammatory responses, which might cooperate in destabilizing neoplastic cells, and making non-MTX tumors less likely to progress and metastasize, thereby, leading to a potentially better outcome among patients who are able to set up appropriately these regulatory homeostatic mechanisms [35].

Based on their global GEP, the sCRC tumors here analyzed could be classified into two major molecular subgroups with significantly different OS rates, using a limited number (i.e. 19) of differentially expressed genes: MTX-like and non-MTX-like sCRC tumors. Thus, higher expression levels of the SPP1, SRPX2, PCOLCE2, miR-122, ADH1B, PRAP1, FBXO32, miR-21, FER1L4 and SALL4 genes, together with lower levels of the miR-135b*, miR-513a-5p, IL13RA2, miR-378e, ADGRG7, ANP32E, CXCL3, BST2 and MOCOS genes were all associated with a significantly shorter OS, both among the whole patient series and when we restricted the analysis to patients with distant (synchronous and metachronous) metastasis. Up-regulation of the SPP1, miR-122, SRPX2, ADH1B, miR-21, SALL4 and PRAP1 genes, as well as down-regulation of miR-378e have been previously associated with an increased tumor cell migration capacity among sCRC [44–46], greater tumor progression potential [45, 47, 48], a metastatic phenotype [10, 45, 48, 49] and inhibition of apoptosis [50, 51]. In contrast, control of tumor cell proliferation by both activation of apoptosis and up-regulation of both DNA repair genes and the inflammatory response, have been associated with higher expression levels of the ANP32E [52], miR-513a [36], IL13RA2 [53] and CXCL3 [54] genes; however, controversial results have been found in other studies for the latter two markers [55, 56]. Similarly, previous studies have also found an association between higher expression levels of FER1L4 and tumor suppressor functions [57], and between up-regulation of the miR-135b and BST2 genes and both metastatic [58] and poor prognosis colorectal tumors [59]. In this regard, Gaedcke *et al.* [60] and Aslam *et al.* [61] have previously reported an association between down-regulation of miR-135b in Dukes’ stage B sCRC patients who developed metastatic disease and a shorter disease-free survival; although these results are fully in line with our observations, they could not be systematically confirmed by others [58, 59]. In turn, the greater BST2 gene expression levels found here among non-MTX tumors, could potentially be more related to the immunomodulatory role of stromal cells expressing BST2 in non-MTX sCRC, rather than the tumor cell-specific expression levels *per se*; this would contribute to explain, at least in part, the apparent discrepant findings in the literature [62]. Such apparent discrepancies, as well as the precise role of other genes which have not been previously associated with sCRC (e.g: the ADGRG7, MOCOS, FBXO32 and PCOLCE2 genes), deserve further investigations. In this regard, integration of the 19-gene prognostic classifier here proposed into avatar mouse models (i.e: patient-derived xenograft models) might provide the opportunity for fast and detailed longitudinal *in vivo* analyses, aimed at establishing efficient models for accurate discrimination between lethal and non-lethal tumors (predictive medicine) at the earliest stages of sCRC tumor development and progression [63]. Of note, similar GEP were observed among MTX tumors that presented distant metastasis already at diagnosis and those that only showed metastatic disease later on during follow-up.

Despite the role of each specific gene, the 19-gene classifier here described showed an independent effect on sCRC patient OS particularly when combined with the lymph node status at diagnosis. Based on these findings, we built a prognostic classification that allowed stratification of sCRC patients into three risk groups with significantly different OS rates, including a standard-risk group of patients with a non-MTX GEP profile, a high-risk group with both a MTX-like GEP and nodal disease (*N* ≥ 1) at diagnosis and an intermediate-prognosis subgroup including 45% of sCRC tumors, who despite sharing non-invasive disease, had a MTX-like GEP at diagnosis with an intermediate median OS of 6 years.

Despite previous studies have described several prognostic associations in sCRC patients for both gene-coding and miRNA-expression signatures, such studies have mostly focused on stage II–IIII sCRC [64–66] and GEP that distinguish between early and advanced TNM stage tumors [67, 68], as well as good- vs. poor-prognosis sCRC patients [3, 49, 69, 70] and GEP associated with (local and/or distant) tumor recurrence [71–74]. Here we evaluated for the first time, the impact of the global coding and non-coding GEP of primary MTX vs. non-MTX tumors (with a long follow-up) on sCRC patient OS. Of note, the 19-gene classifier here defined showed a direct (but limited) overlap with previous prognosis-associated sCRC gene signatures which have also included differential expression of the SPP1 [67–69] FBXO32 [67], miR-21 [65, 66] and miR-135b [15] genes. Such apparent discrepancies might be due to differences in patient selection (i.e: selection of patients at any TNM stage including non-MTX cases with a long follow-up), the specific microarray platforms used and/or the way the prognostically informative genes vs. pathways/networks, have been selected/identified [15, 16].

In summary, here we report a clear association between the overall (coding and non-coding) GEP of primary sCRC tumor cells and their metastatic potential, and provide the basis for further prognostic stratification, already at diagnosis, of sCRC patients undergoing complete tumor resection, and to the identification of new biomarkers for sCRC tumor cell dissemination. The understanding of the differentially de-regulated biological pathways in MTX vs. non-MTX sCRC tumors might contribute in the future to the design of an individualized precision medicine trials based on the reported (bio) markers [75]. However, due to the still limited number of cases analyzed, further prospective validation of the 19-gene classifier and the prognostic classification here proposed in larger, independent series of cases, including higher numbers of sCRC patients at risk of harboring distant metastasis (i.e; TNM stage II/III cases studied at diagnosis) are required.

## MATERIALS AND METHODS

### Patients and samples

Tissue specimens from 48 consecutive sporadic (sCRC) patients who underwent surgical resection of primary tumor tissues (from June 2000 to September 2007) at the Department of Surgery of the University Hospital of Salamanca (Salamanca, Spain), were included in this study, prior to any cytotoxic therapy was given.

In all cases, tumor diagnosis and classification was performed according to the AJCC criteria [76]. Median follow-up at the moment of closing the study was of 103 months (range: 1–172 months). Half of the patients (*n* = 23; 48%) developed liver metastases (Group 1: metastatic sCRC) either during the first 8 months after colorectal surgery (*n* = 16; hereinafter referred as synchronous liver metastasis) or later on during follow-up (*n* = 7; hereinafter referred as metachronous metastasis). The other 25 patients (52%) corresponded to non-metastatic sCRC selected based on the absence of metastatic dissemination after a minimum follow-up of 5 years (Group 2: non-metastatic cases). In addition, non-tumoral colorectal tissue specimens (i.e: normal mucosa) were also collected from 9/48 patients (7 non-metastatic and 2 metastatic cases). Seventeen of 23 metastatic tumors (74%) and all non-metastatic sCRC tumors have been previously reported in the literature [8–10]. Patient clinical, laboratory and follow-up data is summarized in Table 3 and detailed in Supplementary Table 3.

**Table 3:** Clinical and biological characteristics of patients with metastatic (*n* = 23) versus non-metastatic (*n* = 25) sporadic colorectal carcinoma (sCRC) at diagnosis

Variable	Non-metastatic sCRC (*n* = 25)	Metastatic sCRC (*n* = 23)	*p*-value	Total (*n* = 48)
Age (years)^*^	70 (62–77)	66 (61–75)	NS	70 (61–76)
**Gender**				
Female	7 (28%)	7 (30%)	NS	14 (29%)
Male	18 (72%)	16 (70%)		34 (71%)
Tumor size (cm)^*^	5 (4–5)	5 (4–7)	NS	5 (4–6.9)
**Site of primary tumor**				
Left colon	13 (52%)	10 (43%)	.009	23 (48%)
Right colon	9 (36%)	2 (9%)		11 (23%)
Rectum	3 (12%)	11 (48%)		14 (29%)
CEA serum levels (ng/ml)^*^	2.5 (1.3–4.9)	45 (6.8–155)	<.001	5.8 (2.2–45)
ALP serum levels (mg/dl)^*^	123 (72–213)	82 (72–128)	NS	93 (73–174)
**Histopathological grade**				
Well- differentiated	18 (72%)	11 (48%)	NS	29 (61%)
Moderately- differentiated	6 (24%)	10 (43%)		16 (33%)
Poorly- differentiated	1 (4%)	2 (9%)		3 (6%)
**Lymph node involvement**				
N0	25 (100%)	8 (35%)	<.001	33 (69%)
N1	0 (0%)	10 (44%)		10 (21%)
N2	0 (0%)	5 (22%)		5 (10%)
**TNM stage at diagnosis**				
I	7 (28%)	1 (4%)	<.001	8 (17%)
IIA	11 (44%)	3 (13%)		14 (29%)
IIB	7 (28%)	0 (0%)		7 (15%)
IIIB	0 (0%)	5 (22%)		5 (10%)
IIIC	0 (0%)	1 (4%)		1 (2%)
IV	0 (0%)	13 (57%)		13 (27%)
**N. of deaths**	9 (36%)	18 (78%)	.003	27 (56%)
Overall survival (months)^*^	156 (124–155)	41 (17–58)	<.001	103 (31–147)

Results expressed as number of cases (percentage) or ^*^as median (interquartile range, 25th–75th percentile). sCRC: sporadic colorectal cancer; CEA: carcinoembryonic antigen; ALP: alkaline phosphatase; NS: no statistically significant differences found for any group comparisons (*p* ≤ .05).

Colorectal tissue samples not required for diagnostic purposes were collected immediately after surgical resection, snap frozen and stored in OCT at −80°C (Tumor Biobank of the University Hospital of Salamanca, Red de Bancos de Tumores de Castilla y León, Salamanca, Spain), after informed consent was given by each individual prior to entering the study, according to the Declaration of Helsinki. The study was approved by the local ethics committee of the University Hospital of Salamanca (Salamanca, Spain).

Once the histopathological diagnosis had been established, sections from paraffin-embedded tissue samples were cut from three different areas representative of the tumor tissue with >70% tumor cell infiltration by hematoxylin-eosin staining, after excluding stroma-enriched tumor areas. Selection of the neighbour areas of the tumor sections containing ≥70% tumor cells, as well as non-tumoral colorectal tissue samples, for further molecular analyses, was performed on dissected samples stored in OCT.

### RNA extraction and gene expression profiling (GEP) microarray studies

For GEP, sample preparation and array hybridization was performed as described in the Affymetrix GeneChip Expression Analysis Manual (Santa Clara, CA), as previously described [10]. Fluorescence signals from both the Affymetrix PrimeView Human Gene Expression and the microRNA 3.0 Expression arrays hybridized to both tumoral and non-tumoral colorectal total RNA were detected using the GeneChip Scanner 3000 7G (Affymetrix) and data stored as .CEL files (data are publicly available at the GSE81582 data bank).

For data analysis, GEP raw data was normalized with the Robust Multi-array Average algorithm implemented in the Affymetrix R package (v.1.52.0) [77]. The custom Brainarray CDF files were used to link probes in the microarray to genes annotated in the Ensembl database (v.20) [78]. Differentially expressed genes between different groups of tumor samples, as well as between tumoral and non-tumoral tissues, were identified by the supervised two-class unpaired Significance Analysis of Microarray implemented in the siggenes R-package (v.1.46.0). In order to assure a low false discovery rate (FDR) <.05 corrected *p*-values (Benjamini–Hochberg procedure) were used for multiple comparisons. Differentially expressed genes were selected based on an absolute fold change cut-off of ≥2.0. Functional enrichment analysis of deregulated genes associated to molecular (KEGG) pathways was based on simultaneous usage of the GeneCodis [79] and the DIANA-miRPath v.3 [80] software.

In order to identify the best combination of genes to discriminate between the GEP of metastatic (Group 1) and non-metastatic (Group 2) sCRC primary tumors (vs. non-neoplastic colorectal tissues), a four-step strategy was used. In the first step, only those transcripts that were differentially expressed between Group 1 and Group 2 sCRC tissues were selected. In the second step, those genes identified to be differentially expressed in the first step were used to select for the most discriminating genes between the two tumor groups and non-tumoral tissues by using the Leave One Out Cross Validation re-sampling method implemented in the caret R-package (v.6.0-71); for this purpose, the importance of each variable in the model was evaluated using the glmnet method [81] and the best prediction model was obtained after selecting for variables with a contribution ≥45% (overall error rate of 5.3% based on 19 variables). Finally, in order to support the sCRC GEP classifier, unsupervised HCA of samples and genes capable of discriminating between Group 1 and Group 2 sCRC tumors, were implemented in the SIMFIT software based on the specific log_2_ expression signals detected for each gene. Clustering was run using Euclidean distances and the group average linkage method. The best discriminating cut-off for each group-associated gene was assessed by receiver operating characteristic ROC curve analysis (IBM SPSS Corp. v 23; Armonk, NY). The homogeneity of GEP of MTX sCRC tumors with a potentially different metastatic behavior (i.e: metastatic patients with vs. without distant metastasis at diagnosis) were verified prior to any analysis (FDR > .4) and the accuracy of the model to discriminate non-MTX sCRC tumors from those sCRC who showed (distant) metastasis only during follow-up, was measured by using Support Vector Machine algorithms and the Leave One Out Cross Validation re-sampling method implemented in the Babelomics 5.0 package [82].

### Other statistical methods

For categorical variables, the χ^2^ test was used to evaluate the statistical significance of differences observed between groups (IBM SPSS Corp.). Overall survival (OS) curves were plotted according to the Kaplan and Meier method, and the log-rank test (one-sided) was used to establish the statistical significance of differences observed between OS curves (IBM SPSS Corp.). Optimal cut-off values for each variable were calculated using the Cutoff Finder web tool [83] as those values associated with the most significant differences in OS by the one-sided log-rank test. Multivariate analysis of prognostic factors for OS was performed using the Cox stepwise regression (forward selection) model (IBM SPSS Corp.) based on those variables that showed a trend towards significance (*p* < .2) in the univariate analysis. The potential prognostic ability estimate for the Cox proportional hazards model was assessed by the concordance probability (Harrell´s c-index) calculated with the dynpred R-package (version 0.1.2) [84].

## SUPPLEMENTARY MATERIALS FIGURES AND TABLES





## Abbreviations

AJCC: American Joint Committee on Cancer
ALP: alkaline phosphatase
AUC: area under the curve
CEA: carcinoembryonic antigen
ECM: extracellular Matrix
FDR: false discovery rate
GEP: gene expression profile
GnRH: Gonadotropin-releasing hormone
HCA: hierarchical clustering analyses
KEGG: Kyoto Encyclopedia of Genes and Genomes
MTX: metastatic
non-MTX: non-metastatic
(nc)RNA: non-coding RNA
OS: overall survival
ROC: receiver operating characteristic
sCRC: sporadic colorectal cancer
TNM: Tumor Node Metastasis
