# Supplementary material for: Prognostic impact of a novel gene expression profile classifier for the discrimination between metastatic and non-metastatic primary colorectal cancer tumors
Source: Oncotarget. 2017 Nov 21;8(64):107685–700. doi: 10.18632/oncotarget.22591 (PMC5746100; doi:10.18632/oncotarget.22591)
Supplement: Supplementary file 1 [file oncotarget-08-107685-s001.pdf]

Prognostic impact of a novel gene expression profile classifier for the discrimination between metastatic and non-metastatic primary colorectal cancer tumors

SUPPLEMENTARY MATERIALS

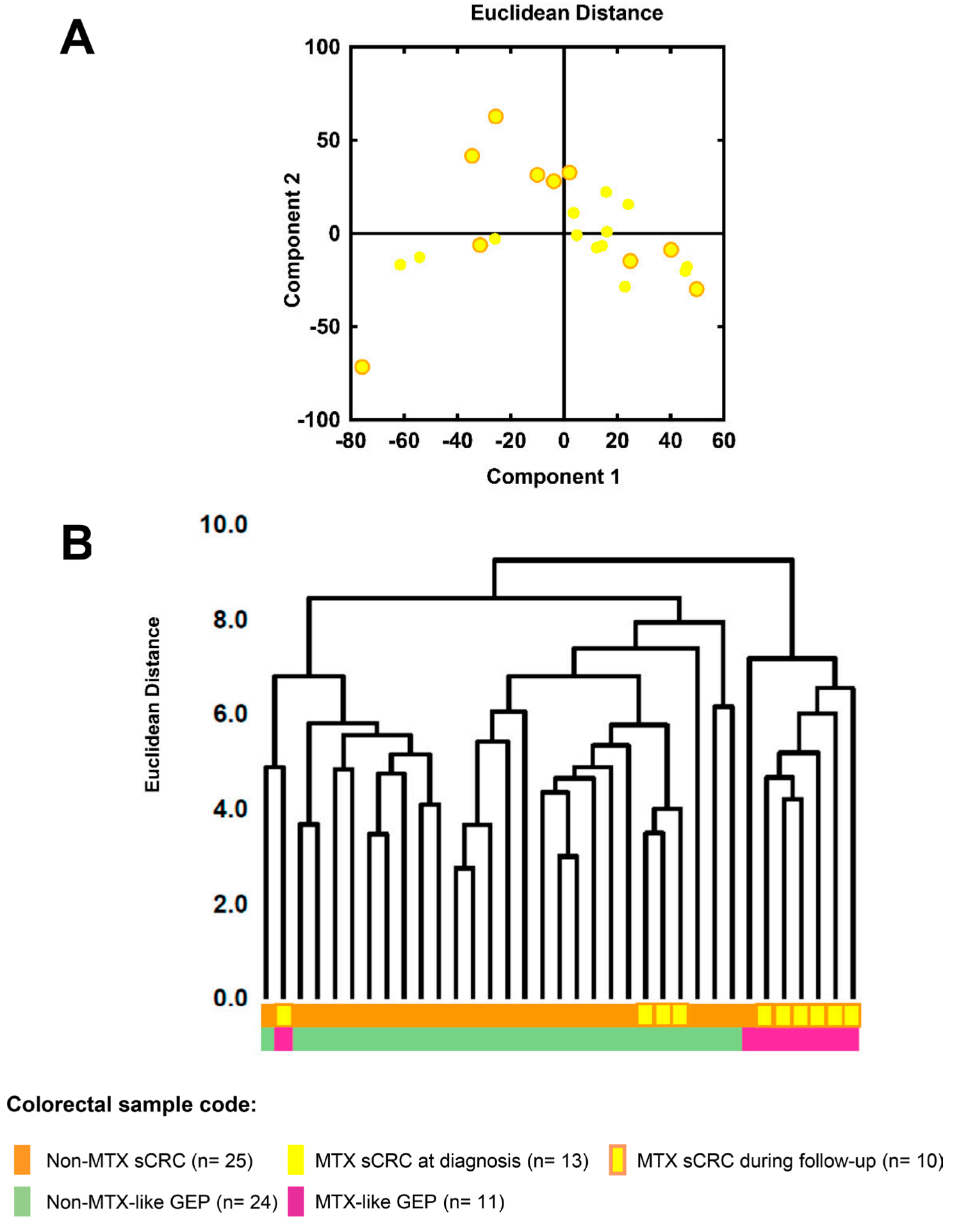

Supplementary Figure 1: GEP of metastatic sCRC tumors which presented liver metastasis at diagnosis vs. follow-up (panel **A**) and validation of the discrimination power of the 19-gene GEP-classifier in sCRC tumors without metastatic dissemination at diagnosis (panel **B**). In panel A, unsupervised multidimensional scaling scatterplot of sCRC patients who presented with liver metastasis at diagnosis (colored yellow; $n = 13$) vs. follow-up (colored yellow with orange frames; $n = 10$) based on the global-coding (mRNA) and non-coding (small nuclear and microRNA)-GEP, while in panel B unsupervised hierarchical clustering analysis of sCRC tumors without metastatic dissemination at diagnosis was used to graphically represent the accuracy (97%) of the expression levels of the 19 genes of the proposed GEP classifier to discriminate non-metastatic sCRC cases (colored orange; $n = 25$) from sCRC which developed metastatic, liver disease during follow-up (colored yellow with orange frames; $n = 10$).

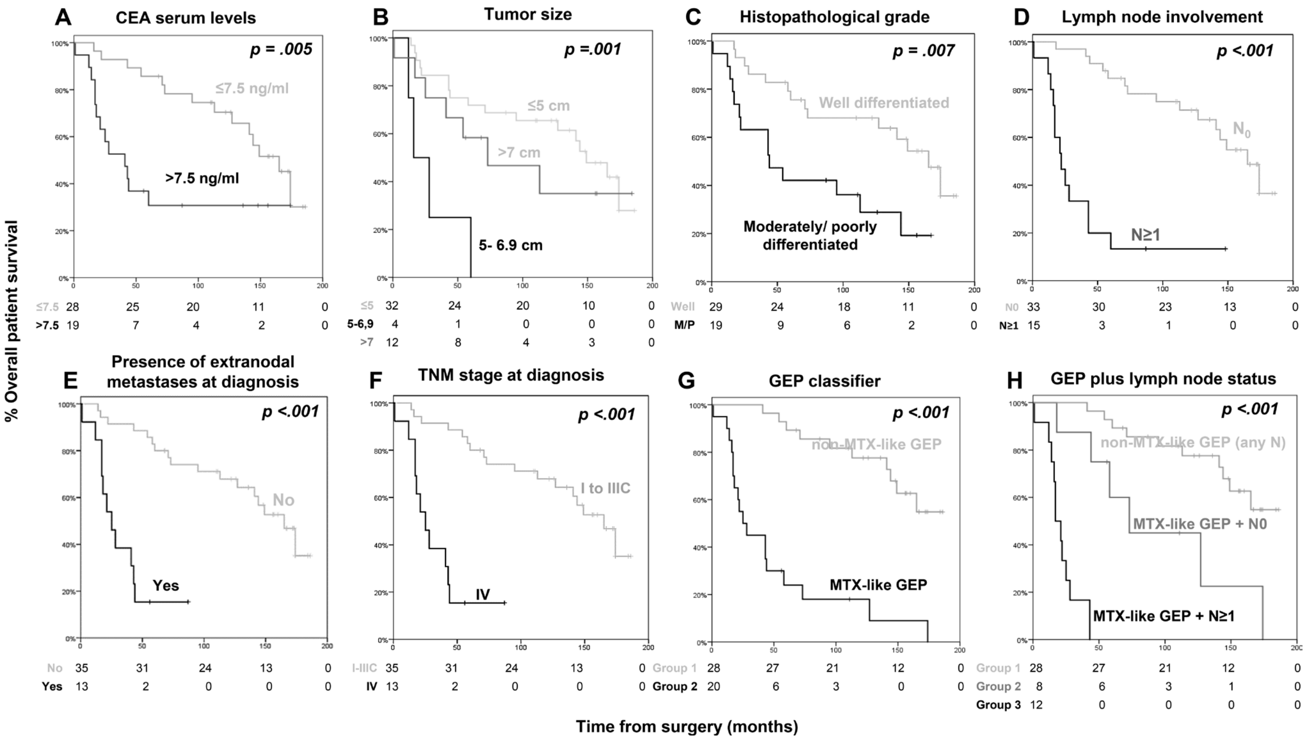

Supplementary Figure 2: Clinical, biological and histopathological characteristics with a significant impact on overall survival of sCRC patients. (**A**) carcinoembryonic antigen serum levels (CEA), (**B**) tumor size, (**C**) histopathological grade, (**D**) lymph node involvement, (**E**) occurrence of distant metastases at diagnosis, (**F**) TNM stage, (**G**) the GEP classifier built on the basis of those 19 coding and non-coding RNA genes that better discriminated metastatic from non-metastatic sCRC at diagnosis, and (**H**) the GEP plus lymph node status (presence ($N \geq 1$) vs. absence (N0) of lymph node infiltration at diagnosis.

Supplementary Table 1: Gene transcripts differentially expressed in primary metastatic vs. non-metastatic sCRC tumors (*n* = 23 vs. *n* = 25, respectively) vs. non- tumoral colorectal tissues (*n* = 9). See Supplementary_Table_1

Supplementary Table 2: Most representative molecular pathways involved in sCRC primary tumors as identified through analysis of the GEP of coding and non-coding RNAs shared by metastatic (*n* = 23) and non-metastatic (*n* = 25) sCRC primary tumors vs. non-tumoral colorectal tissues (*n* = 9)

KEGG functional pathway		Pathway ID code	non-MTX sCRC		MTX sCRC	
			N. of genes (%)*	Corrected *p*-value‡	N. of genes (%)*	Corrected *p*-value‡
**Metabolism and gastrointestinal system**	Fat digestion and absorption	04975	9 (21%)	.001	8 (19%)	.001
	Nitrogen metabolism	00910	7 (32%)	<.001	6 (27%)	.001
	Ether lipid metabolism	00565	7 (21%)	.005	7 (21%)	.002
	Glycerolipid metabolism	00561	8 (17%)	.007	6 (13%)	.02
	Pentose and glucuronate interconversions	00040	5 (24%)	.01	5 (24%)	.006
	Steroid hormone biosynthesis	00140	7 (17%)	.01	6 (14%)	.01
	Metabolism of xenobiotics by cytochrome P450 (1)	00980	8 (14%)	.01	6 (10%)	.04
	Drug metabolism-cytochrome P450	00982	8 (14%)	.01	6 (10%)	.04
	Starch and sucrose metabolism	00500	6 (16%)	.02	6 (16%)	.01
**Intracellular signal transduction**	PPAR signaling pathway	03320	12 (17%)	<.001	8 (11%)	.01
	p53 signaling pathway	04115	11 (17%)	.001	8 (12%)	.01
	TGF-β signaling pathway	04350	10 (13%)	.01	11 (14%)	.001
	MAPK signaling pathway	04010	21 (8%)	.02	21 (8%)	.002
	Calcium signaling pathway	04020	15 (9%)	.03	12 (7%)	.04
**Cell communication, and intercellular signaling molecules and interaction**	Cytokine-cytokine receptor interaction (2)	04060	30 (12%)	<.001	26 (11%)	<.001
	ECM-receptor interaction (1)	04512	13 (16%)	<.001	13 (16%)	<.001
	Gap junction	04540	11 (13%)	.008	9 (11%)	.01
	Tight junction	04530	12 (9%)	.04	10 (8%)	.04
**Tissue homeostasis**	Pancreatic secretion	04972	13 (14%)	.003	14 (15%)	<.001
	Mineral absorption	04978	8 (16%)	.009	6 (12%)	.03
	Aldosterone-regulated sodium reabsorption	04960	6 (15%)	.03	6 (15%)	.01
	Vascular smooth muscle contraction	04270	14 (13%)	.003	11 (10%)	.01
**Cell cycle, growth and death**	Cell cycle	04110	28 (24%)	<.001	20 (17%)	<.001
	Oocyte meiosis	04114	13 (12%)	.007	9 (8%)	.04
	Progesterone-mediated oocyte maturation	04914	10 (12%)	.02	9 (11%)	.01
**Cancer**	Small cell lung cancer	05222	12 (14%)	.003	9 (11%)	.01
	Pathways in cancer	05200	26 (8%)	.009	23 (7%)	.004
	Bladder cancer	05219	7 (18%)	.01	8 (20%)	<.001
**Intercellular transport and catabolism**	Amoebiasis	05146	13 (14%)	.003	14 (15%)	<.001
	Malaria	05144	8 (17%)	.006	6 (13%)	.02
	Phagosome	04145	11 (10%)	.04	9 (8%)	.04
**Immune system**	Chemokine signaling pathway	04062	21 (12%)	<.001	20 (11%)	<.001
	Rheumatoid arthritis	05323	10 (14%)	.009	10 (14%)	.002

*The percentage of genes within a functional category is expressed as the ratio between the number of genes differentially expressed in the GEP assigned to a molecular pathway and the total number of genes which are annotated for that same pathway. ‡*p*-value corrected for multiple hypothesis using the false discovery rate method of Benjamini and Hochberg. KEGG pathway results were obtained from both gene coding data analysis and simultaneous analysis of coding and non-coding gene data for (1) non-metastatic primary sCRC tumors or (2) metastatic sCRC tumors. PPAR: peroxisome proliferator-activated receptors; ECM: extracellular matrix; MAPK: mitogen-activated protein kinase.

Supplementary Table 3: Detailed clinical and biological characteristics of each individual metastatic (*n* = 23) and non-metastatic (*n* = 25) colorectal cancer patient analyzed (*n* = 48)

Case ID	Gender	Age (year)	Site of primary tumor	Histological grade	Primary tumor size (cm)	TNM stage at diagnosis		N. of metastasis	Type of liver metastasis*	CEA serum levels (ng/ml)
1	M	69	Left colon	Well	3.0	T3N0M0	IIA	1	Metachronous	7.6
2	M	57	Left colon	Well	6.0	T3N1M1	IV	>3	Synchronous	30.9
3	M	77	Left colon	Well	5.5	T3N1M0	IIIB	1	Metachronous	244.9
4	F	80	Left colon	Well	3.0	T4N0M1	IV	1	Synchronous	85.3
5	M	64	Left colon	Well	7.0	T3N0M1	IV	>3	Synchronous	256.0
6	M	75	Rectum	Well	4.0	T3N1M0	IIIB	2	Metachronous	589.2
7	F	62	Rectum	Well	7.5	T3N0M1	IV	1	Synchronous	139.0
8	M	63	Rectum	Well	7.0	T3N2M1	IV	1	Synchronous	23.2
9	M	77	Rectum	Well	9.0	T3N1M1	IV	1	Synchronous	58.3
10	M	64	Rectum	Well	7.0	T3N0M0	IIA	1	Metachronous	5.4
11	M	66	Rectum	Well	8.5	T3N0M0	IIA	1	Metachronous	3.7
12	M	76	Right colon	Moderate	5.5	T3N1M1	IV	>3	Synchronous	43.9
13	M	62	Right colon	Moderate	3.0	T3N2M1	IV	3	Synchronous	155.2
14	F	58	Left colon	Moderate	5.0	T4N2M1	IV	>3	Synchronous	501.0
15	F	75	Left colon	Moderate	9.0	T4N1M1	IV	>3	Synchronous	1145.0
16	M	61	Left colon	Moderate	3.0	T2N0M0	I	1	Metachronous	1.2
17	F	76	Rectum	Moderate	2.5	T3N1M0	IIIB	3	Synchronous	149.8
18	F	49	Rectum	Moderate	6.5	T3N1M0	IIIB	2	Synchronous	6.8
19	M	74	Rectum	Moderate	5.0	T4N0M1	IV	>3	Synchronous	110.0
20	M	73	Rectum	Moderate	5.5	T3N1M0	IIIB	1	Synchronous	6.4
21	M	61	Rectum	Moderate	5.0	T3N2M0	IIIC	1	Metachronous	2.3
22	F	48	Left colon	Poor	4.0	T4N2M1	IV	>3	Synchronous	32.9
23	M	72	Left colon	Poor	4.0	T3N1M1	IV	2	Synchronous	45.4
24	M	56	Left colon	Well	5	T3N0M0	IIA	0		18
25	M	69	Left colon	Well	4.5	T4N0M0	IIB	0		9.9
26	M	79	Right colon	Well	5	T3N0M0	IIA	0		13.5
27	F	83	Left colon	Well	4	T2N0M0	I	0		1.3
28	M	76	Rectum	Well	5	T3N0M0	IIA	0		2.3
29	M	77	Left colon	Well	7	T3N0M0	IIA	0		0.6
30	M	70	Left colon	Well	5	T2N0M0	I	0		4.5
31	M	51	Left colon	Well	5	T4N0M0	IIB	0		1.2
32	F	72	Left colon	Well	3	T2N0M0	I	0		1.4
33	F	70	Right colon	Well	2.5	T3N0M0	IIA	0		4.9
34	M	45	Rectum	Well	8	T4N0M0	IIB	0		3.4
35	F	73	Left colon	Well	4.5	T4N0M0	IIB	0		4.9
36	M	74	Right colon	Well	4.5	T3N0M0	IIA	0		2.5
37	M	38	Left colon	Well	5	T3N0M0	IIA	0		1
38	M	61	Rectum	Well	3	T2N0M0	I	0		3.6
39	M	66	Left colon	Well	4	T2N0M0	I	0		3.1
40	M	72	Right colon	Well	4	T4N0M0	IIB	0		2
41	F	57	Right colon	Well	4	T3N0M0	IIA	0		0.8
42	M	77	Right colon	Moderate	8	T3N0M0	IIA	0		2.1
43	M	77	Left colon	Moderate	5	T3N0M0	IIA	0		3.4
44	M	67	Right colon	Moderate	14	T4N0M0	IIB	0		7.3
45	F	77	Right colon	Moderate	5	T3N0M0	IIA	0		6.3
46	M	66	Left colon	Moderate	3.5	T2N0M0	I	0		1.9
47	M	63	Left colon	Moderate	5	T2N0M0	I	0		1.2
48	F	78	Right colon	Poor	7	T4N0M0	IIB	0		1.7

*Synchronous metastasis were defined as these metastasis that were either present already at diagnosis or that developed during the first 8 months after colorectal surgery. M: male; F: female; CEA: carcinoembryonic antigen.
