# Supplementary material for: Prognostic impact of a novel gene expression profile classifier for the discrimination between metastatic and non-metastatic primary colorectal cancer tumors
Source: Oncotarget. 2017 Nov 21;8(64):107685–700. doi: 10.18632/oncotarget.22591 (PMC5746100; doi:10.18632/oncotarget.22591)
Supplement: Supplementary file 2 [file oncotarget-08-107685-s002.docx]

**Supplementary Table 1.** Gene transcripts differentially expressed in primary metastatic vs non-metastatic sCRC tumors (n=23 vs n=25, respectively) vs non- tumoral colorectal tissues (n=9).

| **Gene Symbol** | **Affymetrix ID code** | **MTX vs non-MTX** | |  | **MTX vs non-tumoral** | |  | **non-MTX vs non-tumoral** | |
| --- | --- | --- | --- | --- | --- | --- | --- | --- | --- |
|  |  | **Fold Change** | **FDR** |  | **Fold Change** | **FDR** |  | **Fold Change** | **FDR** |
| ACA10 | ACA10_s_st | -2.15 | 0.034 |  | 3.83 | <0.001 |  | 3.47 | <0.001 |
| ACA21 | ACA21_st | -2.44 | 0.046 |  | 2.09 | 0.044 |  | 3.48 | 0.001 |
| ACA24 | ACA24_s_st | -4.22 | 0.007 |  |  |  |  | -2.18 | <0.001 |
| ACA26 | ACA26_st | -2.01 | 0.030 |  | 3.06 | <0.001 |  | 3.48 | <0.001 |
| ACA52 | ACA52_st | -2.12 | 0.023 |  | 3.01 | <0.001 |  | 3.48 | <0.001 |
| ACA57 | ACA57_st | -2.32 | 0.040 |  |  |  |  | -2.36 | 0.002 |
| ACTA2 | ENSG00000107796_at | 2.30 | 0.034 |  | 6.58 | 0.001 |  | 6.80 | <0.001 |
| ADGRG7 | ENSG00000144820_at | -2.31 | 0.026 |  |  |  |  | 2.14 | 0.036 |
| ADH1B | ENSG00000196616_at | 2.39 | 0.039 |  |  |  |  | 2.20 | 0.021 |
| AEBP1 | ENSG00000106624_at | 2.41 | 0.024 |  |  |  |  | 2.15 | <0.001 |
| AKAP12 | ENSG00000131016_at | 2.51 | 0.032 |  | 9.68 | <0.001 |  | 6.09 | <0.001 |
| ANP32E | ENSG00000143401_at | -2.38 | 0.002 |  | 3.49 | 0.001 |  | 7.65 | <0.001 |
| ASPN | ENSG00000106819_at | 2.15 | 0.038 |  | -3.30 | <0.001 |  | -2.95 | <0.001 |
| BST2 | ENSG00000130303_at | -2.66 | 0.048 |  |  |  |  |  |  |
| CASP1 | ENSG00000137752_at | -2.24 | 0.025 |  |  |  |  | 2.26 | 0.018 |
| CNN1 | ENSG00000130176_at | 2.23 | 0.038 |  | 7.07 | <0.001 |  | 5.85 | <0.001 |
| COMP | ENSG00000105664_at | 2.96 | 0.034 |  | 2.16 | 0.046 |  |  |  |
| CXCL3 | ENSG00000163734_at | -2.60 | 0.015 |  |  |  |  | 2.35 | 0.004 |
| CXCL5 | ENSG00000163735_at | -4.14 | 0.019 |  | 9.91 | <0.001 |  | 9.82 | <0.001 |
| CYP1B1 | ENSG00000138061_at | 2.23 | 0.040 |  | 2.24 | 0.040 |  | 2.15 | 0.029 |
| DES | ENSG00000175084_at | 2.63 | 0.046 |  | 6.48 | <0.001 |  | 6.03 | <0.001 |
| FABP4 | ENSG00000170323_at | 6.06 | 0.029 |  | 3.11 | 0.040 |  | 5.80 | 0.001 |
| FBXO32 | ENSG00000156804_at | 2.10 | 0.008 |  | 13.06 | <0.001 |  | 6.35 | 0.001 |
| FER1L4 | ENSG00000088340_at | 2.06 | 0.050 |  |  |  |  | 2.92 | 0.012 |
| GRP | ENSG00000134443_at | 2.39 | 0.023 |  | -2.58 | 0.001 |  | -2.26 | 0.004 |
| HBII-115 | HBII-115_st | -2.32 | 0.028 |  | 2.89 | 0.001 |  | 3.49 | <0.001 |
| HBII-180A | HBII-180A_x_st | -2.49 | 0.005 |  | 3.49 | <0.001 |  | 3.49 | <0.001 |
| HBII-180C | HBII-180C_x_st | -2.40 | 0.024 |  | 3.57 | 0.001 |  | 3.49 | 0.001 |
| HBII-85-26 | HBII-85-26_st | -5.02 | <0.001 |  | -2.35 | 0.001 |  |  |  |
| HBII-85-6 | HBII-85-6_x_st | -3.30 | 0.001 |  |  |  |  |  |  |
| HMCN1 | ENSG00000143341_at | 2.03 | 0.039 |  |  |  |  | 2.79 | <0.001 |
| hp_hsa-mir-3648 | hp_hsa-mir-3648_st | -2.11 | 0.020 |  | 2.73 | <0.001 |  | 3.52 | <0.001 |
| hp_hsa-mir-3676 | hp_hsa-mir-3676_st | -3.62 | 0.007 |  |  |  |  |  |  |
| hp_hsa-mir-4449 | hp_hsa-mir-4449_st | -2.64 | 0.002 |  | 2.40 | 0.005 |  | 3.53 | <0.001 |
| hp_hsa-mir-4634 | hp_hsa-mir-4634_st | -2.02 | 0.034 |  | 2.65 | 0.015 |  | 3.54 | 0.001 |
| hp_hsa-mir-548ai | hp_hsa-mir-548ai_st | -4.55 | <0.001 |  |  |  |  | 3.61 | <0.001 |
| hsa-let-7f | hsa-let-7f_st | 2.20 | 0.013 |  | 2.30 | 0.006 |  | 3.56 | <0.001 |
| hsa-miR-1180 | hsa-miR-1180_st | -2.01 | 0.041 |  |  |  |  | 2.17 | <0.001 |
| hsa-miR-1202 | hsa-miR-1202_st | -2.20 | 0.023 |  | 4.70 | <0.001 |  | 3.57 | <0.001 |
| hsa-miR-122 | hsa-miR-122_st | 2.39 | 0.001 |  | -2.24 | 0.005 |  | -2.18 | 0.005 |
| hsa-miR-1225-5p | hsa-miR-1225-5p_st | -2.46 | 0.030 |  | 2.80 | <0.001 |  | 3.58 | <0.001 |
| hsa-miR-1231 | hsa-miR-1231_st | -2.49 | 0.012 |  | 2.19 | <0.001 |  | 3.59 | <0.001 |
| hsa-miR-1285 | hsa-miR-1285_st | -2.14 | 0.012 |  | 2.61 | 0.002 |  | 3.59 | <0.001 |
| hsa-miR-1307 | hsa-miR-1307_st | -3.99 | 0.005 |  | 2.81 | <0.001 |  | 3.62 | <0.001 |
| hsa-miR-130b | hsa-miR-130b_st | -3.43 | 0.006 |  | 2.09 | 0.010 |  | 3.64 | <0.001 |
| hsa-miR-135b-star | hsa-miR-135b-star_st | -2.06 | 0.010 |  | 4.26 | <0.001 |  | 3.67 | <0.001 |
| hsa-miR-1469 | hsa-miR-1469_st | -2.04 | 0.008 |  | 2.26 | <0.001 |  | 3.69 | <0.001 |
| hsa-miR-146a | hsa-miR-146a_st | -2.51 | 0.018 |  | 2.32 | 0.001 |  | 2.66 | <0.001 |
| hsa-miR-150-star | hsa-miR-150-star_st | -2.65 | 0.011 |  | 15.90 | <0.001 |  | 3.69 | 0.015 |
| hsa-miR-17-star | hsa-miR-17-star_st | -2.65 | 0.018 |  | 2.48 | 0.011 |  | 3.74 | <0.001 |
| hsa-miR-18a-star | hsa-miR-18a-star_st | -2.24 | 0.021 |  | 4.15 | <0.001 |  | 3.76 | <0.001 |
| hsa-miR-1909 | hsa-miR-1909_st | -2.12 | 0.036 |  | 3.24 | <0.001 |  | 3.79 | <0.001 |
| hsa-miR-1910 | hsa-miR-1910_st | -2.30 | 0.004 |  | 2.50 | 0.039 |  | 3.80 | 0.002 |
| hsa-miR-194-star | hsa-miR-194-star_st | -3.06 | 0.013 |  |  |  |  |  |  |
| hsa-miR-19a | hsa-miR-19a_st | -2.54 | 0.010 |  |  |  |  | 2.05 | 0.001 |
| hsa-miR-19b | hsa-miR-19b_st | -2.36 | 0.006 |  |  |  |  | 3.10 | 0.001 |
| hsa-miR-200a-star | hsa-miR-200a-star_st | -3.06 | 0.013 |  |  |  |  | 2.38 | <0.001 |
| hsa-miR-21 | hsa-miR-21_st | 2.08 | 0.001 |  |  |  |  | 2.03 | 0.008 |
| hsa-miR-23a-star | hsa-miR-23a-star_st | -2.74 | 0.004 |  | 2.74 | <0.001 |  | 3.85 | <0.001 |
| hsa-miR-25-star | hsa-miR-25-star_st | -3.55 | 0.001 |  | 3.27 | <0.001 |  | 3.85 | <0.001 |
| hsa-miR-27a-star | hsa-miR-27a-star_st | -4.40 | 0.002 |  | 2.32 | 0.035 |  | 3.87 | 0.001 |
| hsa-miR-3156-5p | hsa-miR-3156-5p_st | -2.65 | 0.005 |  | 2.24 | 0.021 |  | 3.88 | 0.001 |
| hsa-miR-3175 | hsa-miR-3175_st | -3.57 | 0.027 |  | 4.05 | <0.001 |  | 3.90 | <0.001 |
| hsa-miR-3176 | hsa-miR-3176_st | -3.01 | 0.003 |  |  |  |  | 2.23 | 0.003 |
| hsa-miR-3178 | hsa-miR-3178_st | -2.71 | 0.044 |  | 2.78 | <0.001 |  | 3.91 | <0.001 |
| hsa-miR-3188 | hsa-miR-3188_st | -3.02 | 0.011 |  | 2.37 | 0.018 |  | 3.95 | <0.001 |
| hsa-miR-3195 | hsa-miR-3195_st | -2.87 | 0.019 |  | 2.95 | <0.001 |  | 3.97 | <0.001 |
| hsa-miR-324-5p | hsa-miR-324-5p_st | -2.09 | 0.041 |  |  |  |  |  |  |
| hsa-miR-330-3p | hsa-miR-330-3p_st | -2.53 | 0.012 |  | 3.69 | 0.002 |  | 5.34 | <0.001 |
| hsa-miR-339-3p | hsa-miR-339-3p_st | -2.66 | 0.013 |  |  |  |  | 2.11 | <0.001 |
| hsa-miR-3613-3p | hsa-miR-3613-3p_st | -4.04 | 0.001 |  | 8.10 | <0.001 |  | 3.98 | <0.001 |
| hsa-miR-3615 | hsa-miR-3615_st | -2.21 | 0.002 |  | 3.20 | 0.001 |  | 3.98 | <0.001 |
| hsa-miR-362-5p | hsa-miR-362-5p_st | -3.99 | 0.016 |  | 2.68 | 0.005 |  | 4.00 | <0.001 |
| hsa-miR-3663-3p | hsa-miR-3663-3p_st | -2.32 | 0.013 |  | 2.68 | <0.001 |  | 4.06 | <0.001 |
| hsa-miR-374b | hsa-miR-374b_st | 2.34 | 0.029 |  |  |  |  | 3.79 | 0.001 |
| hsa-miR-378 | hsa-miR-378_st | -2.86 | 0.012 |  |  |  |  | 2.23 | 0.017 |
| hsa-miR-378c | hsa-miR-378c_st | -3.33 | 0.014 |  |  |  |  | 3.17 | 0.009 |
| hsa-miR-378d | hsa-miR-378d_st | -2.85 | 0.016 |  |  |  |  | 2.17 | 0.017 |
| hsa-miR-378e | hsa-miR-378e_st | -2.22 | 0.008 |  |  |  |  | 2.15 | 0.008 |
| hsa-miR-378f | hsa-miR-378f_st | -2.97 | 0.014 |  |  |  |  | 3.30 | 0.013 |
| hsa-miR-378g | hsa-miR-378g_st | -2.59 | 0.020 |  |  |  |  |  |  |
| hsa-miR-378i | hsa-miR-378i_st | -2.91 | 0.013 |  |  |  |  | 2.36 | 0.013 |
| hsa-miR-422a | hsa-miR-422a_st | -3.36 | 0.007 |  |  |  |  | 2.49 | 0.001 |
| hsa-miR-423-3p | hsa-miR-423-3p_st | -2.11 | 0.036 |  |  |  |  | 2.09 | 0.002 |
| hsa-miR-425-star | hsa-miR-425-star_st | -2.20 | 0.034 |  |  |  |  | 2.01 | 0.022 |
| hsa-miR-4286 | hsa-miR-4286_st | -3.51 | 0.003 |  |  |  |  | 2.37 | 0.001 |
| hsa-miR-4322 | hsa-miR-4322_st | -2.02 | 0.017 |  | 4.52 | <0.001 |  | 4.08 | <0.001 |
| hsa-miR-4329 | hsa-miR-4329_st | 2.05 | 0.032 |  | 4.40 | <0.001 |  | 4.09 | <0.001 |
| hsa-miR-4440 | hsa-miR-4440_st | -2.68 | 0.023 |  | 2.74 | 0.010 |  | 4.13 | <0.001 |
| hsa-miR-4443 | hsa-miR-4443_st | -3.07 | 0.031 |  | 2.35 | 0.001 |  | 4.17 | <0.001 |
| hsa-miR-4449 | hsa-miR-4449_st | -3.83 | 0.005 |  | 2.82 | 0.005 |  | 4.18 | <0.001 |
| hsa-miR-4454 | hsa-miR-4454_st | -3.29 | 0.004 |  |  |  |  | 2.07 | 0.001 |
| hsa-miR-4492 | hsa-miR-4492_st | -2.89 | 0.014 |  | 2.65 | 0.016 |  | 4.20 | <0.001 |
| hsa-miR-4505 | hsa-miR-4505_st | -2.94 | 0.016 |  | 2.72 | 0.001 |  | 4.23 | <0.001 |
| hsa-miR-4507 | hsa-miR-4507_st | -2.61 | 0.011 |  | 4.45 | <0.001 |  | 4.27 | <0.001 |
| hsa-miR-4532 | hsa-miR-4532_st | -2.69 | 0.035 |  |  |  |  | 2.18 | 0.001 |
| hsa-miR-4634 | hsa-miR-4634_st | -2.79 | 0.025 |  | 3.25 | 0.001 |  | 4.27 | <0.001 |
| hsa-miR-4651 | hsa-miR-4651_st | -2.30 | 0.010 |  | 3.29 | <0.001 |  | 4.31 | <0.001 |
| hsa-miR-4665-5p | hsa-miR-4665-5p_st | -2.04 | 0.021 |  | 3.20 | 0.022 |  | 4.34 | 0.004 |
| hsa-miR-4668-5p | hsa-miR-4668-5p_st | -4.36 | 0.001 |  |  |  |  | 2.14 | 0.001 |
| hsa-miR-4674 | hsa-miR-4674_st | -2.75 | 0.010 |  | 2.60 | 0.037 |  | 4.34 | 0.001 |
| hsa-miR-4690-5p | hsa-miR-4690-5p_st | -2.33 | 0.012 |  | 5.52 | 0.005 |  | 4.39 | 0.012 |
| hsa-miR-4707-5p | hsa-miR-4707-5p_st | -2.47 | 0.021 |  | 2.69 | 0.004 |  | 4.40 | <0.001 |
| hsa-miR-4721 | hsa-miR-4721_st | -2.39 | 0.005 |  | 8.73 | <0.001 |  | 4.48 | <0.001 |
| hsa-miR-4734 | hsa-miR-4734_st | -3.44 | 0.015 |  | 4.45 | 0.002 |  | 4.52 | 0.001 |
| hsa-miR-4741 | hsa-miR-4741_st | -2.37 | 0.020 |  | 3.12 | 0.008 |  | 4.53 | 0.001 |
| hsa-miR-4745-5p | hsa-miR-4745-5p_st | -2.57 | 0.012 |  | 3.28 | <0.001 |  | 4.58 | <0.001 |
| hsa-miR-4767 | hsa-miR-4767_st | -2.13 | 0.005 |  | 3.24 | <0.001 |  | 4.61 | <0.001 |
| hsa-miR-4786-5p | hsa-miR-4786-5p_st | -2.66 | 0.005 |  |  |  |  | 2.30 | 0.001 |
| hsa-miR-4793-3p | hsa-miR-4793-3p_st | -2.57 | 0.043 |  | 2.88 | 0.008 |  | 4.63 | <0.001 |
| hsa-miR-500a | hsa-miR-500a_st | -2.39 | 0.030 |  | 5.66 | <0.001 |  | 4.63 | <0.001 |
| hsa-miR-501-3p | hsa-miR-501-3p_st | -2.06 | 0.038 |  |  |  |  | 2.54 | <0.001 |
| hsa-miR-501-5p | hsa-miR-501-5p_st | -2.43 | 0.027 |  | 4.37 | <0.001 |  | 4.66 | <0.001 |
| hsa-miR-513a-5p | hsa-miR-513a-5p_st | -2.09 | 0.015 |  |  |  |  | 2.32 | 0.001 |
| hsa-miR-532-5p | hsa-miR-532-5p_st | -2.91 | 0.021 |  | 3.40 | <0.001 |  | 4.66 | <0.001 |
| hsa-miR-652 | hsa-miR-652_st | -2.42 | 0.048 |  |  |  |  | 2.03 | <0.001 |
| hsa-miR-663 | hsa-miR-663_st | -2.59 | 0.048 |  | 6.45 | 0.001 |  | 4.69 | 0.004 |
| hsa-miR-671-5p | hsa-miR-671-5p_st | -2.10 | 0.004 |  |  |  |  |  |  |
| hsa-miR-675 | hsa-miR-675_st | -2.16 | 0.014 |  | 2.74 | 0.010 |  | 4.72 | <0.001 |
| hsa-miR-720 | hsa-miR-720_st | -4.02 | 0.014 |  |  |  |  | 2.02 | <0.001 |
| hsa-miR-92b-star | hsa-miR-92b-star_st | -2.10 | 0.020 |  | 3.27 | 0.005 |  | 4.75 | <0.001 |
| hsa-miR-933 | hsa-miR-933_st | -2.04 | 0.008 |  |  |  |  | 2.09 | 0.001 |
| hsa-miR-939 | hsa-miR-939_st | -2.12 | 0.035 |  | 6.62 | <0.001 |  | 4.78 | 0.001 |
| hsa-miR-93-star | hsa-miR-93-star_st | -3.24 | 0.008 |  | 3.80 | 0.001 |  | 4.81 | <0.001 |
| IL13RA2 | ENSG00000123496_at | -2.13 | 0.012 |  | 4.43 | 0.004 |  | 7.42 | <0.001 |
| ITGA7 | ENSG00000135424_at | 2.18 | 0.018 |  | 9.81 | <0.001 |  | 7.07 | <0.001 |
| LCN2 | ENSG00000148346_at | -2.79 | 0.034 |  | 15.39 | <0.001 |  | 9.02 | <0.001 |
| MAP1B | ENSG00000131711_at | 2.13 | 0.004 |  | 4.32 | <0.001 |  | 7.06 | <0.001 |
| mgh18S-121 | mgh18S-121_st | -2.59 | 0.025 |  |  |  |  | 2.13 | <0.001 |
| mgh28S-2411 | mgh28S-2411_st | -2.56 | 0.032 |  | 4.95 | 0.040 |  | 4.83 | 0.044 |
| MGP | ENSG00000111341_at | 2.69 | 0.012 |  | 5.52 | <0.001 |  | 6.82 | <0.001 |
| MMP10 | ENSG00000166670_at | -2.35 | 0.012 |  | 8.37 | <0.001 |  | 8.18 | <0.001 |
| MMP3 | ENSG00000149968_at | -3.78 | 0.017 |  |  |  |  | 2.56 | 0.009 |
| MOCOS | ENSG00000075643_at | -3.10 | 0.001 |  | 6.66 | <0.001 |  | 8.27 | <0.001 |
| MYH11 | ENSG00000133392_at | 2.69 | 0.013 |  | 2.06 | 0.026 |  | 2.13 | 0.006 |
| MYL9 | ENSG00000101335_at | 2.17 | 0.008 |  | 6.35 | <0.001 |  | 6.52 | <0.001 |
| OIP5 | ENSG00000104147_at | -2.01 | 0.024 |  | 7.45 | <0.001 |  | 8.84 | <0.001 |
| PBK | ENSG00000168078_at | -2.32 | 0.005 |  | 5.28 | <0.001 |  | 8.68 | <0.001 |
| PCOLCE2 | ENSG00000163710_at | 2.51 | 0.007 |  | 3.85 | <0.001 |  | 6.34 | <0.001 |
| PIGR | ENSG00000162896_at | -4.20 | 0.041 |  |  |  |  | 2.01 | 0.021 |
| PRAP1 | ENSG00000165828_at | 2.15 | 0.032 |  | 4.11 | 0.015 |  | 6.05 | <0.001 |
| REG1A | ENSG00000115386_at | -9.45 | 0.016 |  | 4.69 | 0.018 |  | 14.47 | <0.001 |
| REG3A | ENSG00000172016_at | -8.46 | 0.006 |  | 10.84 | <0.001 |  | 10.48 | <0.001 |
| SALL4 | ENSG00000101115_at | 2.02 | 0.005 |  |  |  |  | 2.46 | 0.012 |
| SFRP4 | ENSG00000106483_at | 3.18 | 0.012 |  |  |  |  | 2.37 | 0.032 |
| SLIT2 | ENSG00000145147_at | 2.11 | 0.003 |  | 4.15 | <0.001 |  | 7.24 | <0.001 |
| SNORA24 | ENSG00000207130_s_st | -3.22 | 0.010 |  | 2.01 | 0.009 |  |  |  |
| SNORA24 | ENSG00000206903_s_st | -3.31 | 0.008 |  |  |  |  | 3.07 | 0.002 |
| SNORA38 | ENSG00000201042_s_st | -2.48 | 0.013 |  | 4.83 | 0.002 |  | 7.43 | <0.001 |
| SNORA38B | SNORA38B_s_st | -2.87 | 0.019 |  |  |  |  | -2.26 | 0.022 |
| SNORA38B | SNORA38B_s_st | -3.54 | 0.008 |  |  |  |  | -2.35 | 0.001 |
| SNORA38B | SNORA38B_s_st | -2.09 | 0.043 |  | 3.72 | 0.003 |  | 4.83 | <0.001 |
| SNORD116 | ENSG00000202498_x_st | -2.18 | 0.006 |  |  |  |  | 2.37 | <0.001 |
| SPP1 | ENSG00000118785_at | 3.65 | 0.039 |  |  |  |  | 2.16 | 0.040 |
| SRPX2 | ENSG00000102359_at | 2.66 | 0.017 |  |  |  |  | 2.12 | 0.028 |
| TAGLN | ENSG00000149591_at | 2.76 | 0.027 |  | 3.56 | <0.001 |  | 6.04 | <0.001 |
| THBS4 | ENSG00000113296_at | 2.66 | 0.021 |  |  |  |  | -3.40 | 0.003 |
| TYMS | ENSG00000176890_at | -2.11 | 0.003 |  | 8.91 | <0.001 |  | 7.73 | <0.001 |
| U104 | U104_st | -2.22 | 0.005 |  |  |  |  | -4.85 | 0.023 |
| U105B | U105B_st | -2.30 | 0.018 |  |  |  |  | -3.61 | <0.001 |
| U23 | U23_st | -2.89 | 0.001 |  | 2.76 | 0.004 |  | 4.88 | <0.001 |
| U23 | U23_st | -2.91 | 0.010 |  | 3.13 | 0.003 |  | 5.08 | <0.001 |
| U28 | U28_st | -2.62 | 0.009 |  | 4.18 | <0.001 |  | 5.10 | <0.001 |
| U3 | U3_s_st | -2.44 | 0.010 |  | 3.57 | 0.030 |  | 5.14 | 0.004 |
| U31 | U31_st | -3.10 | 0.014 |  | 3.40 | 0.001 |  | 5.21 | <0.001 |
| U3-2 | U3-2_s_st | -2.19 | 0.020 |  | 4.52 | 0.029 |  | 5.25 | 0.014 |
| U3-3 | U3-3_s_st | -2.29 | 0.016 |  | 3.70 | 0.008 |  | 5.25 | 0.001 |
| U34 | U34_st | -2.30 | 0.024 |  |  |  |  | 2.08 | <0.001 |
| U3-4 | U3-4_s_st | -2.65 | 0.009 |  | 3.05 | 0.026 |  | 5.27 | 0.001 |
| U36B | U36B_st | -2.03 | 0.023 |  | 3.53 | 0.005 |  | 5.33 | <0.001 |
| U37 | U37_st | -2.34 | 0.016 |  | 3.03 | <0.001 |  | 5.37 | <0.001 |
| U38A | U38A_x_st | -3.03 | 0.012 |  | -3.28 | 0.004 |  | -3.75 | <0.001 |
| U38A | U38A_x_st | -3.16 | 0.014 |  | 3.09 | 0.019 |  | 5.39 | 0.001 |
| U43 | U43_st | -2.10 | 0.043 |  | 3.32 | 0.001 |  | 5.41 | <0.001 |
| U43 | U43_st | -2.16 | 0.023 |  | 3.57 | 0.006 |  | 5.43 | <0.001 |
| U48 | U48_st | -2.79 | 0.019 |  | 3.63 | <0.001 |  | 5.44 | <0.001 |
| U50B | U50B_st | -2.28 | 0.026 |  | -3.15 | 0.001 |  |  |  |
| U58C | U58C_x_st | -2.26 | 0.008 |  |  |  |  |  |  |
| U68 | U68_st | -2.07 | 0.043 |  | 7.53 | <0.001 |  | 5.50 | <0.001 |
| U68 | U68_st | -2.37 | 0.014 |  | 4.34 | 0.006 |  | 5.50 | 0.001 |
| U74 | U74_x_st | -2.49 | 0.018 |  | 4.12 | <0.001 |  | 5.56 | <0.001 |
| U75 | U75_x_st | -2.00 | 0.023 |  | 3.38 | 0.005 |  | 5.68 | <0.001 |
| U75 | U75_x_st | -2.46 | 0.038 |  | 2.91 | 0.034 |  | 5.71 | 0.001 |
| U99 | U99_st | -2.22 | 0.005 |  | 8.14 | <0.001 |  | 5.76 | <0.001 |
| ZBTB20 | ENSG00000181722_at | 2.00 | 0.001 |  |  |  |  | 2.79 | <0.001 |
| ZEB1 | ENSG00000148516_at | 2.00 | 0.008 |  | 8.27 | <0.001 |  | 7.24 | <0.001 |
| ZG16B | ENSG00000162078_at | -2.39 | 0.040 |  |  |  |  | 2.22 | 0.006 |

Only genes with an FDR<.05 for at least one comparisons are shown; MTX: metastatic colorectal cancer; non-MTX: non-metastatic colorectal cancer; hsa-miR: human micro-RNA. FDR: false discovery rate.
